# Genomic Mapping of Splicing-Related Genes Identify Amplifications in *LSM1*, *CLNS1A,* and *ILF2* in Luminal Breast Cancer

**DOI:** 10.3390/cancers13164118

**Published:** 2021-08-16

**Authors:** María del Mar Noblejas-López, Igor López-Cade, Jesús Fuentes-Antrás, Gonzalo Fernández-Hinojal, Ada Esteban-Sánchez, Aránzazu Manzano, José Ángel García-Sáenz, Pedro Pérez-Segura, Miguel De La Hoya, Atanasio Pandiella, Balázs Győrffy, Vanesa García-Barberán, Alberto Ocaña

**Affiliations:** 1 Translational Oncology Laboratory, Translational Research Unit, Albacete University Hospital, 02008 Albacete, Spain; mariadelmar.noblejas@uclm.es; 2 Centro Regional de Investigaciones Biomédicas, Castilla-La Mancha University (CRIB-UCLM), 02008 Albacete, Spain; 3 Molecular Oncology Laboratory, Hospital Clínico San Carlos (HCSC), Instituto de Investigación Sanitaria San Carlos (IdISSC), 28040 Madrid, Spain; ilopez.7@alumni.unav.es (I.L.-C.); ada.esteban@salud.madrid.org (A.E.-S.); miguel.hoya@salud.madrid.org (M.D.L.H.); 4 Experimental Therapeutics Unit, Hospital Clínico San Carlos (HCSC), Instituto de Investigación Sanitaria San Carlos (IdISSC), 28040 Madrid, Spain; jfuentesa@salud.madrid.org (J.F.-A.); gfernandezh@salud.madrid.org (G.F.-H.); aranzazu.manzano@salud.madrid.org (A.M.); 5 Centro de Investigación Biomédica en Red en Oncología (CIBERONC), 28029 Madrid, Spain; 6 Medical Oncology Department, Hospital Clínico San Carlos (HCSC), Instituto de Investigación Sanitaria San Carlos (IdISSC), 28040 Madrid, Spain; jagarcia.hcsc@salud.madrid.org (J.Á.G.-S.); pedro.perez@salud.madrid.org (P.P.-S.); 7 Instituto de Biología Molecular y Celular del Cáncer (IBMCC-CIC), Instituto de Investigación Biomédica de Salamanca (IBSAL), Consejo Superior de Investigaciones Científicas (CSIC) and CIBERONC, 37007 Salamanca, Spain; atanasio@usal.es; 8 Department of Bioinformatics, Semmelweis University, H-1094 Budapest, Hungary; gyorffy.balazs@ttk.mta.hu; 9 2nd Department of Pediatrics, Semmelweis University, H-1094 Budapest, Hungary; 10 TTK Lendület Cancer Biomarker Research Group, Institute of Enzymology, H-1117 Budapest, Hungary

**Keywords:** splicing pathway, luminal breast cancer, BET inhibitors

## Abstract

**Simple Summary:**

The alternative splicing (AS) process is highly relevant, affecting most of the hallmarks of cancer, such as proliferation, angiogenesis, and metastasis. Our study evaluated alterations in 304 splicing-related genes and their prognosis value in breast cancer patients. Amplifications in *CLNS1A*, *LSM1*, and *ILF2* genes in luminal patients were significantly associated with poor outcome. Downregulation of these genes in luminal cell lines showed an antiproliferative effect. Pharmacological modulation of transcription and RNA regulation is key for the optimal development of therapeutic strategies against key proteins. Administration of a BET inhibitor and BET-PROTAC reduced the expression of these identified genes and displayed a significant antiproliferative effect on these cell models. In conclusion, we describe novel splicing genes amplified in luminal breast tumors that are associated with detrimental prognosis and can be modulated pharmacologically. It opens the door for further studies confirming the effect of these genes in patients treated with BET inhibitors.

**Abstract:**

Alternative splicing is an essential biological process, which increases the diversity and complexity of the human transcriptome. In our study, 304 splicing pathway-related genes were evaluated in tumors from breast cancer patients (TCGA dataset). A high number of alterations were detected, including mutations and copy number alterations (CNAs), although mutations were less frequently present compared with CNAs. In the four molecular subtypes, 14 common splice genes showed high level amplification in >5% of patients. Certain genes were only amplified in specific breast cancer subtypes. Most altered genes in each molecular subtype clustered to a few chromosomal regions. In the Luminal subtype, amplifications of *LSM1*, *CLNS1A*, and *ILF2* showed a strong significant association with prognosis. An even more robust association with OS and RFS was observed when expression of these three genes was combined. Inhibition of *LSM1*, *CLNS1A*, and *ILF2*, using siRNA in MCF7 and T47D cells, showed a decrease in cell proliferation. The mRNA expression of these genes was reduced by treatment with BET inhibitors, a family of epigenetic modulators. We map the presence of splicing-related genes in breast cancer, describing three novel genes, *LSM1*, *CLNS1A*, and *ILF2*, that have an oncogenic role and can be modulated with BET inhibitors.

## 1. Introduction

The RNA splicing process regulates gene expression in eukaryotic cells through a complex process in which introns are removed from precursor RNAs (pre-mRNAs) and consecutive exons are precisely joined together to produce mature mRNAs, with the final goal of maintaining mature transcripts to guarantee a successful translation process [1]. The alternative splicing process (AS) is the way in which exons or portions of exons or non-coding regions within a pre-mRNA transcript are differentially joined or skipped, resulting in multiple protein isoforms being encoded by a single gene [1]. Alternative splicing (AS) contributes to transcriptome (and proteome) diversity in development- and tissue-regulated pathways, as well as in response to multiple physiological signals [2]. Remarkably, large-scale proteomic studies suggest that many predicted alternative transcripts are not translated into proteins, so the exact contribution of AS to protein diversity is currently under dispute [3,4]. On top of that, some authors have suggested a role for AS in buffering mutational consequences [5], and mounting evidence indicates that AS coupled to nonsense-mediated decay is a major post-transcriptional regulator of gene expression [6,7]. Five major types of AS have been described: exon skipping, mutually exclusive exons, intron retention, and alternative 5′or 3′ splice site [8]. The AS process is carried out by the spliceosome and consists of four stages: assembly, activation, catalysis or splicing, and disassembly. In each specific stage of a splicing cycle, different spliceosome subcomplexes are involved (pre-B, B, Bact, B*, C, C*, P, and ILS complexes), which are composed of small nuclear ribonucleoproteins (snRNPs) and non-snRNPs splicing factors [9]. AS is a highly regulated process, with five snRNPs and over 300 non-snRNP proteins identified as recruited to the spliceosome at these specific stages [10].

Changes due to AS can affect the translation rate and the functional role of the mRNA. AS can act on different cellular and biological processes or be involved in tissue specificity, developmental states, or disease conditions, such as cancer [11]. It has a relevant role in cancer development and maintenance, affecting most of the hallmarks of cancer [12,13]. In addition, it can be involved in cancer relapse or resistance to different treatment modalities [12]. Thus, specific isoforms have been identified promoting and supporting neoplastic transformation and tumor development. In a variety of tumor types, AS has been linked to up-regulation of oncogenes, participating in different processes of tumor development, including angiogenesis, cell division, altered metabolism, proliferation, or metastasis [10,14]. In addition, they can also contribute to the deregulation of several non-oncogenic vulnerabilities that are also relevant in the initiation and maintenance of the oncogenic transformation [12].

Alterations in the AS machinery have been identified in different human tumors, and they can affect a network of downstream splicing targets. Using high throughput methodologies, Seiler et al. have described somatic mutations in 119 splicing factors in 33 tumor types, bladder carcinoma and uveal melanoma being those with higher frequencies [15]. Moreover, mutations in splicing factors appear to be mutually exclusive within a tumor, which might indicate that co-occurrence of these mutations may be lethal [15].

Specifically in breast cancer, AS affects major breast cancer-related proteins, such as the estrogen receptor (ER), BRCA1, and BRCA2, among others [16]. Thus, disequilibrium between ERα66 and ERα36 induce abnormal proliferation, and high levels of ERα36 can cause resistance to Tamoxifen [16]. Alterations in components of the regulatory splicing machinery have been described in breast cancer. For example, SF3B1 is involved in the 3′-SS recognition and is one of the most commonly mutated genes with a higher frequency in the metastatic setting [17]. Mutant SF3B1 produces aberrant splicing, inducing metabolic reprogramming [18]. In addition, AS has been described to have a role in drug resistance. For instance, it has been described that, in carriers of BRCA1 exon 11 premature termination codon variants, tumors upregulate exon 11 skipping to produce isoforms that retain residual activity, contributing to PARPi resistance [19]. Overexpression of SF3B1 and SF3B3 are associated with tamoxifen and fulvestrant resistance, and inhibition of another splicing factors, such as ZRANB2 and SYF2, reduces resistance to doxorubicin in breast cancer cells [20,21]. On the other hand, SRSF4 induces apoptosis in cancer cells, in combination with platinum agents [22].

In our study, alterations in 304 splicing factors were evaluated in breast cancer patients using several large datasets. We found high frequency of amplification in *CLNS1A*, *LSM1*, and *ILF2* in Luminal tumors, with a significant association with poor prognosis. Despite the limited information about these genes, they have been associated with oncogenic processes and resistance to treatments. IFL2 deregulation has been related to an aberrant RNA splicing pattern, mainly deregulated skipped exons in genes involved in DNA repair [23]. LSM1 is included in the heteroheptameric complex LSM1-7, which initiates mRNA decay [24]. CLNS1A acts as a Sm chaperone, recruiting Sm proteins to the PRMT5 complex [25]. In our study, genomic regulation of these genes, with a reduction of their expression, decreased proliferation of luminal tumor cells. In addition, treatment with epigenetic modulators, such as the Bromodomain and extraterminal (BET) family of inhibitors, reduced the expression level of these genes, leading to cell growth reduction.

## 2. Materials and Methods

### 2.1. Data Collection and Processing

Processed TCGA (The Cancer Genome Atlas) PanCancer dataset was downloaded through cBioportal (www.cbioportal.org; accessed on 4 December 2019). This dataset contains whole exome sequencing and RNA-Seq data from patients with breast invasive carcinoma, consisting of 696 Luminal, 78 HER2 positive, and 171 Basal tumors and their matched normal tissues. WES data was used to explore CNAs and mutations in 304 splicing factor genes. Splicing related genes were collected from four sources: HUGO Gene Nomenclature Committee and the studies of Hegele et al. [26], Wan et al. [9], and Koedoot et al. [20]. Only somatic non-silent mutations in splicing factor genes were considered (missense, premature termination codon, and IVS+-1,2). Somatic non-silent mutations in splicing factor genes were only considered. In the PanCancer dataset, identification of somatic single nucleotide variations was performed using Mutect. CNAs were assessed as deviations in the tumor sample from the paired normal tissue sample using GISTIC 2.0. GISTIC 2.0 identifies regions significantly amplified or deleted and lists genes found in each “wide peak” region [27]. Value +/− 2 indicates high-level thresholds for amplifications/deletions, respectively, and those with +/− 1 exceed the low-level thresholds but not the high-level thresholds. In addition, the Metabric dataset (www.cbioportal.org; accessed on 4 December 2019) was used to validate results of identified genes with a high level of amplification in >5% of tumors.

### 2.2. Outcome Analysis

The relationship between gene expression levels and patient clinical prognosis in terms of relapse-free survival (RFS) and overall survival (OS) was evaluated using the Kaplan–Meier Plotter platform, as described previously [28,29] (accessed on 6 June 2020). This tool used gene expression and survival data from 7830 breast tumors (sources include GEO, EGA and TCGA). Samples were split into two groups using the best threshold as the cutoff (auto select best cutoff). When testing multiple genes, the analysis was performed using the mean expression. Patients above the threshold were labelled as “high” expressing, while patients below the threshold were labelled as “low” expressing. The two groups were compared using Cox survival analysis. The prognostic value of the identified signature (containing *LSM1*, *CLSN1A,* and *ILF2*) was validated using the TCGA project.

The correlation between CNAs and patient clinical outcome was analyzed using the Genotype-2-Outcome platform (accessed on 8 January 2021) [30]. This tool links genotype to clinical outcome by utilizing next generation sequencing and gene chip data of 6697 breast cancer patients. It allows the association with prognosis of a specific transcriptomic signature linked to an altered gene, by classifying patients according to the average expression of significant genes designated as a surrogate metagene of its alteration status. The median expression values for different transcripts are used as a cut-off to discriminate “high” and “low” expression cohorts, which are compared using a Cox survival analysis. To identify factors independently associated with OS and RFS, a multivariate analysis (Cox proportional risk regression model) was performed.

### 2.3. Cell Culture and Compounds

MCF7 and T47D cells (American Type Culture Collection, Manassas, VA, USA) were cultured in DMEM (Sigma-Aldrich, Saint Louis, MO, USA) containing 10% fetal bovine serum with 100 U/mL penicillin, 100 μg/mL streptomycin, and 2 mM L-glutamine, and cells were maintained at 37 °C in a 5% CO_2_ atmosphere. The BET inhibitor JQ1 was purchased from Tocris Bioscience, and BET-PROTAC MZ1 was purchased from Selleckchem (Houston, TX, USA).

### 2.4. Small Interfering RNA

siRNA oligonucleotides (Sigma-Aldrich, Saint Louis, MO, USA) were transfected into cells using Lipofectamine RNAiMax protocol (Thermo Fisher Scientific, Rockford, IL, USA) at a final concentration of 20 nM. References: siLSM1(EHU121391), siCLNS1A (EHU147241), and siILF2 (EHU084311). siGFP (EHUEGFP) was used as the negative control of transfection. Briefly, cells were transfected (~80% of confluency), and after 24 h, cells were reseeded for validation experiments.

### 2.5. Quantitative Reverse-Transcription PCR

Total RNA was collected from cells using the RNeasy Mini Kit (Qiagen, Hilden, Germany) according to the manufacturer’s instructions. Determination of concentration and purity were measured using a NanoDrop ND-1000 spectrophotometer (Thermo Fisher Scientific, Rockford, IL, USA), and then 1 μg of total RNA was reverse transcribed using the RevertAid H Minus first-strand cDNA synthesis kit (Thermo Fisher Scientific) in a thermal cycler (Bio-Rad, Hercules, CA, USA) under the following reaction conditions: 65 °C for 5 min, 42 °C for 60 min, and 70 °C for 10 min. cDNAs were then subjected to real-time PCR analysis using Fast SYBR Green master mix on the StepOnePlus Real-Time PCR system (Applied Biosystems) according to the manufacturer’s instructions. Primer sequences used were as follows: h-LSM1 F: TTCCTCGAGGGATTTTTGTG, h-LSM1 R: TTCTCTGCTTCCAGCTTGGT, h-CLNS1A F: TCGGCACTGGTACCCTTTAC, h-CLNS1A R: AATGGTGGGGTATTCCAGTG, h-ILF2 F: GCTCCAGGGACATTTGAAGT, h-ILF2 R: CAGCCACATTGTGTCCTGTAG, h-18S F: GAGGATGAGGTGGAACGTGT, h-18S R: TCTTCAGTCGCTCCAGGTCT. An initial step was performed at 95 °C for 5 min, followed by 40 cycles of 95 °C for 15 s, and finished at 60 °C for 1 min. Each sample was analyzed in triplicates, and cycle threshold (Ct) values of transcripts were determined using StepOne Software v.2.1. Ct values were calculated using the 18Sas reference. Untreated control cells were used as the control to calculate the Ct value and determine the X-fold mRNA expression.

### 2.6. Proliferation MTT Assays

Cell proliferation was measured using MTT reagent (3-(4, 5-dimethylthiazol-2-yl)-2, 5 diphenyltetrazolium bromide) (Sigma-Aldrich). MTT reduction by mitochondria of living cells generate formazan accumulates.

For evaluated siRNAs antiproliferative effect, MCF7 and T47D cells (5000/well, 48-multiwell plates) were seeded after siRNAs transfection during 24, 48, and 120 h.

For antiproliferative drugs validation, MCF7 and T47D cells (5000/well, 48-multiwell plates) were seeded. After, they were treated with increased doses of JQ1 and MZ1 for 72 h. Later medium was replaced with red-phenol free DMEM containing MTT (0.5 mg/mL) and incubated for 45 min at 37 °C. After, medium was removed and dimethylsulfoxide (DMSO) (Thermo Fisher Scientific) was used for dissolved formazan accumulates. Absorbance (A555 nm–A690 nm) was recorded in a multiwell plate reader (BMG labtech, Ortenberg, Germany).

### 2.7. Growth Studies

To compare the growth between siRNAs-transfected cells and siGFP-transfected cells (control), proliferation rate was studied by cell count. Cell lines were cultured at a density of 50,000 cells in 6-well. At the times of 24 and 48 h, cells were trypsinized and counted. Images was performed at 48 h using inverted microscope (10×).

### 2.8. Cell Cycle Assay

siRNAs-transfected cells (MCF7 and T47D) were collected and fixed in ethanol (70%, cold) for 30 min. Cell pellets were washed in PBS+2% BSA and incubated in the dark for 1 h at 4 °C with Propidium iodide/RNAse staining solution (Immunostep).

### 2.9. Statistical Analysis

We used the student’s *t*-test unpaired for independent samples. The level of significance was considered 95%; therefore, *p* values lower than 0.05 were considered statistically significant: * *p* < 0.05; ** *p* < 0.01, and *** *p* < 0.001. Statistics and representations were made with statistical software GraphPad Prism 7.0. All results (unless indicated) are presented as the mean ± SEM of three independent experiments, each of them performed in triplicate.

## 3. Results

### 3.1. Mutations in Splicing-Related Genes

**Figure 1:**
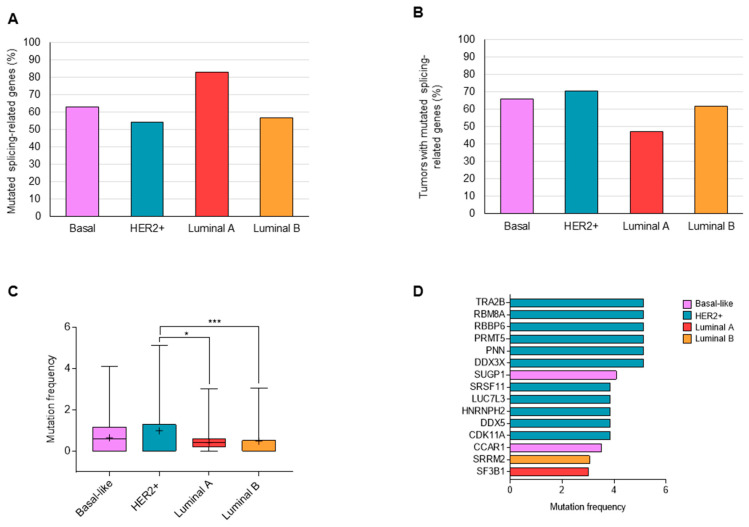
Percentage of splicing-related genes showing mutations in each molecular subtype (**A**). Percentage of tumors with at least one mutated splicing-related gene (**B**). Frequency of non-synonymous mutations in splicing-related genes in each molecular subtype (**C**). List of splicing-related genes mutated in >3% of tumors (**D**). *: *p* < 0.05; ***: *p* < 0.001.

Alterations in 304 splicing-related genes (Supplementary Table S1) were analyzed in 945 breast cancer patients (499 Luminal A, 197 Luminal B, 171 Basal, and 78 HER2+ samples) using the Breast Invasive PanCancer Atlas Dataset, as described in the materials and methods section. Non-synonymous mutations were detected in 278 genes, with 525 tumors showing at least one altered splicing-related gene (Supplementary Table S2, Supplementary Figure S1). When patients were classified based on molecular subtypes, several differences were observed in the distribution of the identified genes. Regarding the 278 genes with presence of mutations, the number of altered genes were 231 (83.1%) for Luminal A, 157 (56.5%) for Luminal B, 175 (62.9%) for Basal, and 150 (54%) for HER2+ subtype (Figure 1A). Tumors with modifications in any of these genes were observed in a higher percentage in HER2+ (70.5%) and Basal (66.1%) compared with the Luminal A and B subtypes (47.1% and 61.9%, respectively) (Figure 1B). When the frequency of tumors with alterations in each gene were evaluated, HER2+ and the basal subtype population showed splicing-related genes with a higher percentage of alterations (Figure 1C; Supplementary Table S2). In the four molecular subtypes, no gene was detected to be mutated in more than 6% of tumors. Splicing genes with mutations in >3% of tumors are displayed in Figure 1C and mainly belonged to the HER2+ and Basal subgroups (Figure 1D).

### 3.2. Copy Number Alterations (CNAs) in Splicing-Related Genes

**Figure 2:**
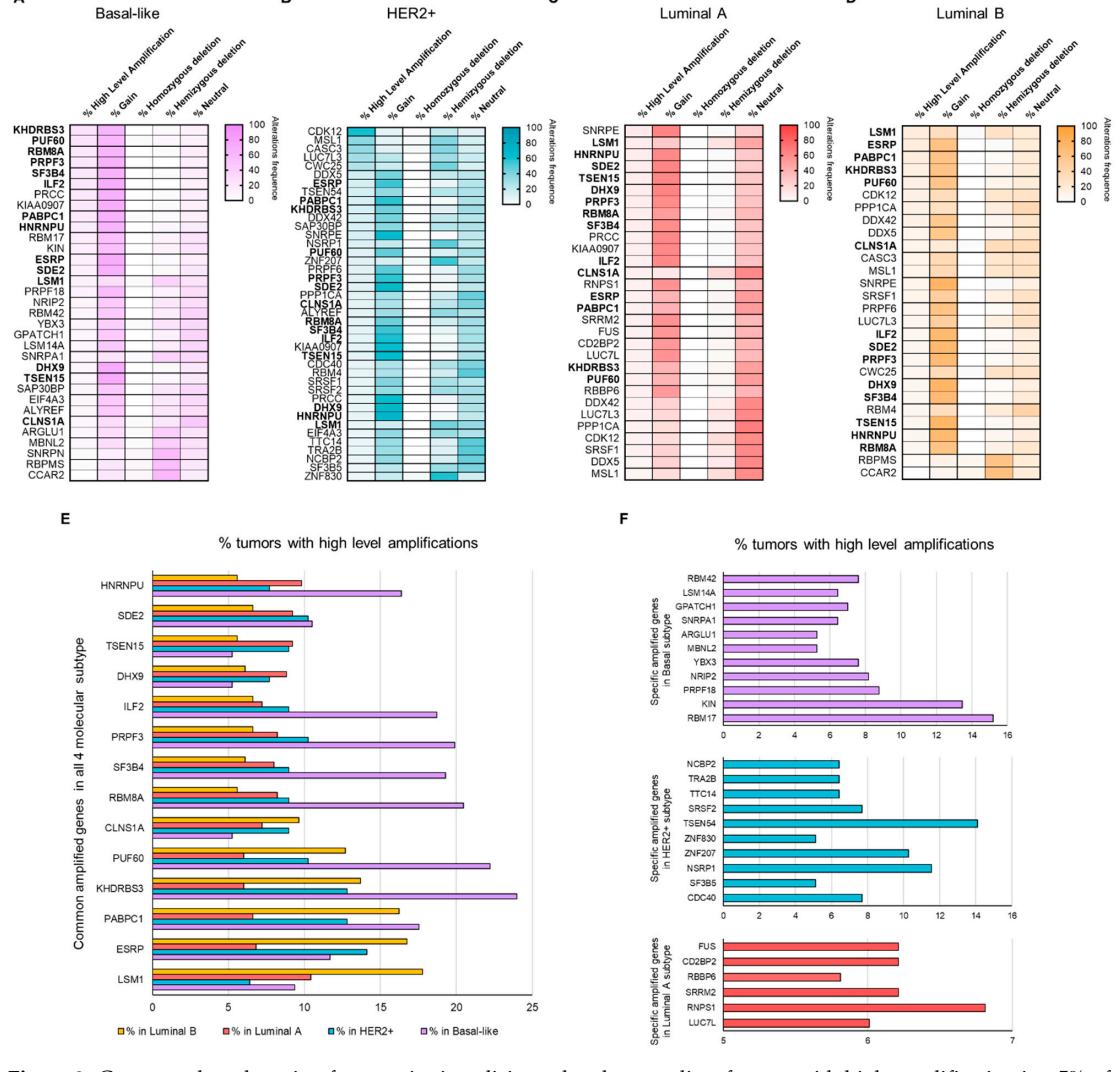
Copy number alteration frequencies in splicing-related genes: list of genes with high amplification in >5% of tumors for Basal (**A**), HER2+ (**B**), Luminal A (**C**), and Luminal B (**D**) molecular subtypes. In total, 14 common splicing-related genes showed high level amplification in >5% of tumors, shown in bold font. Percentage of tumors with presence of amplifications in >5% of tumors in splicing-related genes both common in all molecular subtypes (**E**) and specific in each subtype (**F**).

The TCGA PanCancer series also includes putative copy-number data [31]. Thus, we evaluated the following changes in splicing-related genes: homozygous or hemizygous deletions, no change, gain, and high level of amplification. In this large series of breast cancer patients, we found information about 301 genes from those identified. High level of amplification (GISTIC thresholded CN value of +2) in any of these genes were detected in a high percentage of tumors (HER2+: 87.2%; Basal: 81.3%; Luminal A: 51.1%; and Luminal B: 67.5%). Considering only those genes in regions with homozygous deletion or a high level of amplification in >5% of patients, we found 61 altered splicing-related genes (58 amplified and 3 loss). Regarding the molecular subtypes, 33 (10.9%) splicing-related genes were altered in the Basal subtype (30 amplified and 3 loss), 41 (13.6%) amplified in HER2+, 30 (9.9%) amplified in Luminal A, and 28 (9.3%) altered in Luminal B (26 amplified and 2 loss) (Figure 2A–D, respectively). Therefore, a large number of genes showed higher frequencies of CNAs versus mutations (only 6 genes with mutations in >5% of patients, Figure 1D). In the four molecular subtypes, 14 common splicing-related genes showed a high level of amplification in >5% of tumors (Figure 2E). On the other hand, certain genes were amplified only in specific subtypes (Figure 2F). A complete list of genes is displayed in the Supplementary Table S3.

**Figure 3:**
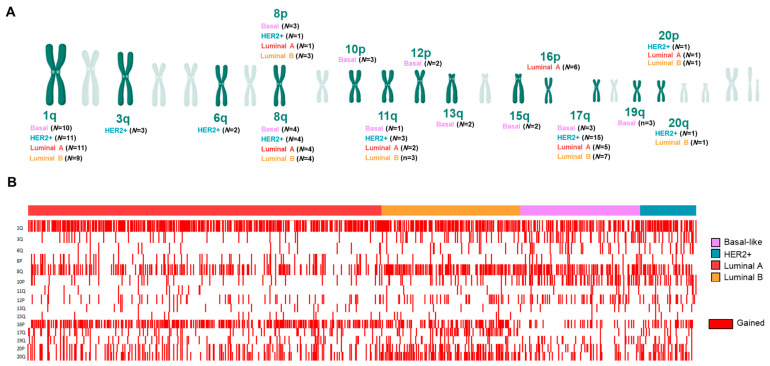
Number of splicing-related genes with high amplification in >5% of patients by chromosome location (**A**) (Created with BioRender.com (accessed on 4 December 2019)). Tumors with chromosomal gain (red) in each molecular subtype (**B**).

With this information, we next aimed to explore the chromosome location of splicing-related genes with a high level of amplification or homozygous deletion. Interestingly, most altered splicing-related genes in each molecular subtype were distributed in a few chromosome regions: 1q, 8q, and 17q, as shown in Figure 3A. In total, 12 out of 14 common amplified genes were located in 1q and 8q. Different altered regions were specific for each subtype: (a) 10p, 12p 13q, 15q, and 19q for Basal; (b) 6q, 17q, and 3q for HER2+; (c) all genes in the 16p region for Luminal A; and (d) no one for Luminal B (Figure 3A; Supplementary Table S3). Copy-number gain in these regions is represented in Figure 3B.

### 3.3. Associations of Splicing-Related Genes with Clinical Outcome in Patients

**Figure 4:**
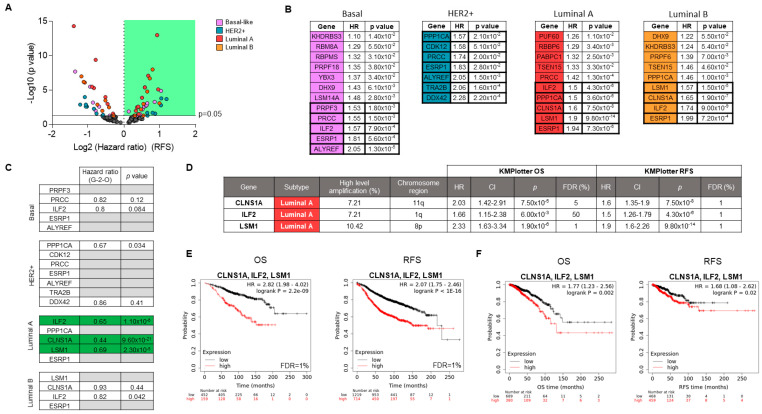
Prognosis value for splicing-related genes with CNAs. Splicing-related genes (only showed those genes with high amplification in >5% of patients) with higher prognostic value based on hazard ratio and *p* values (**A**). List of genes showing significant association between expression levels and detrimental prognosis in RFS (KM Plotter software was used) (**B**). Prognostic value (OS) of selected genes (based on: *p* < 0.002, HR > 1.5; FDR < 5 from KM Plotter) was confirmed using genotype-2-outcome web-server (**C**). Summary of outcome results obtained to *LSM1*, *CLNS1A,* and *ILF2* in the Luminal A subtype (**D**). Kaplan-Meier plots (OS and RFS) for the combination of these three genes using KM Plotter software (**E**) and their validation in another Luminal A cohort (TCGA project) (**F**).

To identify which of the identified genes could have a role in cancer progression, we intended to link the described data with patient clinical outcome. To do so, we used published transcriptomic microarray data, as described elsewhere [32]. The prognostic value of the high amplified genes (with a cutoff of >5% of tumors) were analyzed in a dataset of 6234 breast cancer patients (Figure 4A). CNA frequencies in identified genes were validated in an additional dataset (Supplementary Figure S2). Frequencies of high level of amplified genes were correlated between both datasets. Alterations in splicing-related genes were most frequently observed in the HER2+ and Basal-like subtypes. However, as there were few patients in these subtypes, it was not possible to establish the prognostic value for most amplified genes. Despite the low number of patients in this subgroup, several genes showed association with RFS and OS (Figure 4B and Supplementary Table S4). We focused on those genes, with a clear impact on survival by using an arbitrary selection based on statistical outcome relevance and low false discovery rate (FDR) (*p* < 0.002, Hazard Ratio (HR) > 1.5; FDR < 5). For Luminal A, high expression of *ESRP1*, *LSM1*, *CLNS1A*, *ILF2,* and *PPP1CA* showed a clear association with detrimental OS and RFS (Figure 4B). In the same way, high levels of these first four genes were associated with a poor prognostic in the Luminal B subtype. In the Luminal series, CNAs were significant associated with expression levels in these genes (Supplementary Figure S3).

Next, to confirm the prognostic role of the alterations described before, we used a transcriptomic fingerprint of the amplified splicing-related genes by using the genotype-2-outcome (Figure 4C). With this approach, we can obtain the clinical outcome of a gene signature associated with a specific genomic alteration, including gene amplification, as described in the materials and methods section. Thus, the transcriptomic fingerprint associated with the amplification of *LSM1*, *CLNS1A,* and *ILF2* showed a strong association with survival (Supplementary Figure S4). The transcripts included in the signatures associated with the CNA gain of *LSM1*, *CLNS1A,* and *ILF2* are displayed in Supplementary Table S5.

In Figure 4D, we summarized the prognostic value (RFS and OS), percentage of amplification, subtype, and chromosome location of the identified genes. In addition, a more robust association with OS and RFS was observed when expression of these three genes was combined together (Figure 4E). Finally, the prognostic role of the identified signature in the Luminal A subtype was confirmed in a validation cohort, confirming the reproducibility of the findings described before (TCGA dataset; Figure 4F). Univariate and multivariate COX regression analysis showed that the combination of LSM1, CLNS1A, and ILF2 was a clear, independent prognostic factor, mainly with OS (Supplementary Table S6).

### 3.4. Genomic Down-Regulation of LSM1, CLNS1A, and ILF2 Reduces Cell Proliferation

**Figure 5:**
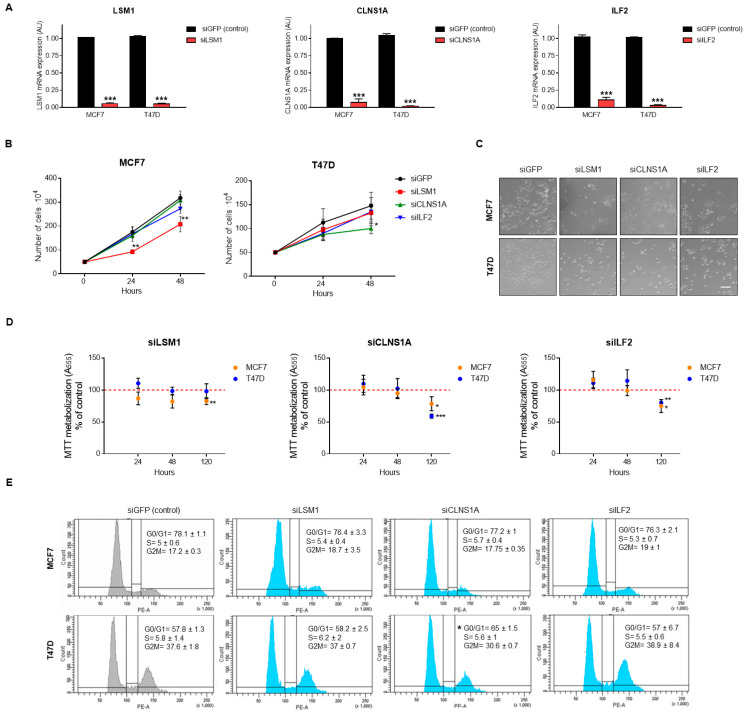
Splicing-related genes genomic inhibition by siRNA and pharmacological inhibition by BET inhibitor and PROTAC. (**A**). *LSM1*, *CLNS1A,* and *ILF2* mRNA expression in MCF7 and T47D luminal A breasts cancer cells after transfection with siRNAs. Cells were transfected using lipofectamine reagent and 24 h later were reseeded. After 24 h, (48 h post-transfection), cells were collected, RNA was extracted, and qPCR was performed. siGFP was used as the control of transfection. (**B**). Transfected cells were seeded (50,000 cells 6-well plate and were counted after 24, 48 and 120 h). (**C**). Images obtained by inverted microscope of transfected cells after 48 h. (**D**). Antiproliferative effect of siRNA evaluated by MTT assays after 24, 48, and 120 h. (**E**). Changes in cell cycle phases after genomic inhibition (representative plot of two independent experiments is shown). Scale bar = 100 μm. * *p* < 0.05; ** *p* < 0.01; *** *p* < 0.001.

To validate *LSM1*, *CLNS1A,* and *ILF2* dependency in Luminal breast cancer cells lines, mRNA expression of these genes was downregulated by using small interfering RNA (siRNA). *LSM1*, *CLNS1A,* and *ILF2* downregulation in MCF7 and T47D efficiently reduced gene expression, as shown in Figure 5A. Cell growth (Figure 5B,C) and cell proliferation, evaluated as MTT metabolization (Figure 5D), was reduced after siRNA knockdown of the mentioned splicing genes. Growth reduction was observed clearer with gene interfering of *LSM1* in MCF7 cells and *CLNS1A* in T47D. The antiproliferative effect produced after gene inhibition evaluated as a metabolization of MTT was significantly observed after 120 h, with no differences at a shorter time. To explore how the mechanism for genomic down-regulation of *LSM1*, *CLNS1A,* and *ILF2* inhibits cell proliferation, we performed cell cycle analysis using propidium iodure. No major changes were observed in cell cycle phases, only *CLNS1A* down-regulation showed a G0/G1 arrest in T47D cells in accordance with previous findings (Figure 5E).

### 3.5. BET Inhibitors Reduce the Expression of LSM1, CLNS1A, and ILF2

**Figure 6:**
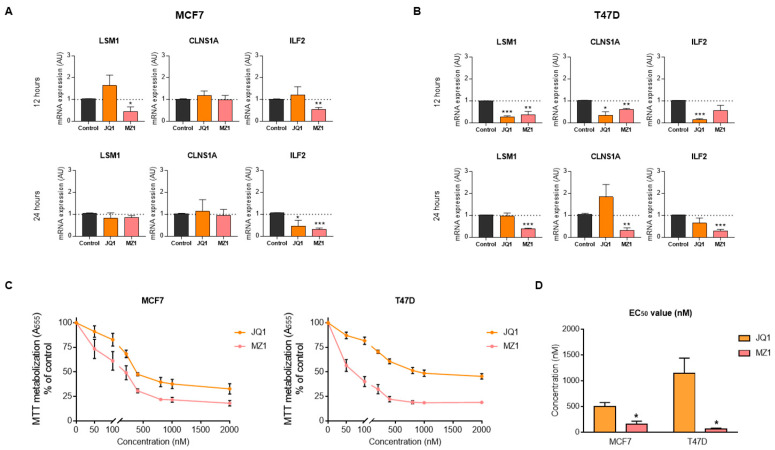
Pharmacological inhibition of splicing-related genes by BET inhibitor and PROTAC. *LSM1*, *CLNS1A*, and *ILF2* mRNA expression in MCF7 (**A**) and T47D (**B**) luminal A breast cancer cell lines after 12 h and 24 h JQ1 and MZ1 exposure. Cell viability evaluated by MTT assays for MCF7 (left) and T47D (right) cells treated with increasing doses of JQ1 and MZ1 (**C**). JQ1, MZ1, and EC50 doses in luminal A cell lines (**D**). * *p* < 0.05; ** *p* < 0.01; *** *p* < 0.001.

Epigenetic agents can modulate the expression of genes that have a role in transcription and maturation [33]. With this in mind, we explored the effect of Bromo and Extra terminal domain (BET) inhibitors and BET derivatives, such as BET-Proteolysis targeting chimeras (PROTAC), on the expression of the identified genes. Administration of the BET inhibitor (JQ1) and BET-PROTAC (MZ1) produced a reduction of the gene expressions of *LSM1*, *CLNS1A,* and *ILF2*. In MCF7 cells, *ILF2* was downregulated with MZ1 treatment after 12 and 24 h of administration. Moreover, *LSM1* was downregulated with MZ1 after 12 h (Figure 6A). In T47D cells, after 12 h of treatment, these three genes were downregulated by both JQ1 and MZ1. This effect was maintained at 24 h of treatment for MZ1, but not for JQ1, suggesting that the PROTAC had a more prolonged effect (Figure 6B). Following these findings, we explored their effect on cell growth. We observed that JQ1 and MZ1 displayed an antiproliferative effect in Luminal cells lines (Figure 6C). EC50 values showed that MZ1 PROTAC was more potent than the inhibitor JQ1 (Figure 6D). In summary, these findings confirm the modulation of the expression of these three genes by JQ1 and MZ1 and the pharmacological effect of these agents on cell proliferation.

## 4. Discussion

In the present article, we characterize the presence and role of genomic alterations in splicing genes in breast cancer. Splicing is a biological process that permits transcriptional diversity and redundancy of molecular functions, allowing the integrity of key cellular activities [34]. Transcriptional regulation by splicing has been involved in the control of different biological tasks from DNA damage, to cell survival, or stemness, among others [13]. In this context, several genes implicated in splicing have been described in cancer, leading to the promotion of different oncogenic properties. For instance, some known factors, such as SRSF1, have been described as overexpressed in cancer, leading to malignant transformation by an alternative splicing of genes involved in proliferation and apoptosis [35]. Other examples include RBM39 in Acute Myeloid Leukemia or RBM11 in glioblastoma cells, among others [36,37]. In breast cancer, mutations in *SF3B1* are more frequently observed in the metastatic setting, and its potential role in the regulation of protein degradation or metabolism is known [17,18]. In addition, overexpression of *SF3B1* and *SF3B3* has been associated with resistance to hormone therapy, and others, such as *ZRANB2* and *SYF2*, to chemotherapy, particularly for doxorubicin [20,21]. Taking this background into consideration, the identification of deregulated genes involved in splicing and the understanding of their role in cancer is a main objective, with the final goal of designing or implementing therapeutic strategies to reduce their presence.

In our study, we analyzed a set of genes involved in splicing in breast cancer. Genomic modifications of splicing proteins were highly presented in breast cancer, the HER2 subtype being the most common tumor (70.5%), with the less frequency presence observed in the Luminal A subtype (47.1%). Although mutations in splicing genes have been widely reported [15], in our study, no specific gene was mutated in more than 6% of the tumors. On the other hand, when CNAs in our splicing-related gene lists were evaluated, 61 of them were altered in >5% of patients. In a similar way to mutations, the HER2 subtype showed a higher number of altered genes compared with the other groups. This really demonstrates that mutations are less frequently observed than other structural alterations, and the splicing pathway is mainly altered in the HER2 subtype compared with the other breast subtypes. Nevertheless, 14 common splicing-related genes showed high-level amplification in >5% of patients in the four molecular groups, most of them located in 1q and 8q chromosome regions.

The next step in our study was to select those altered splicing-related genes with a role in patient clinical outcome. The results were not conclusive for the HER2 subtype due to the small number of patients. Regarding the Luminal subtype, we identified three genes with clear association with poor prognosis: *ILF2*, *LSM1,* and *CLNS1A*. Although prognosis value cannot be evaluated in HER2 and Basal subtypes, these three genes were also detected as amplified in tumors of these molecular subtypes. Moreover, when the presence of CNAs in our selected genes was analyzed in different tumor types (GDC Data Portal; 67 primary sites), breast cancer was one of most frequently amplified for *LSM1*, *CLNS1A,* and *ILF2* (Supplementary Figure S5). IFL2 has been described as implicated in the RNA splicing regulation of crucial effectors involved in DNA damage response [23]. In addition, overexpression of this gene mediated resistance to DNA damaging agents [23]. Of note, *IFL2* is located at the 8p chromosome, where other genes with a particular oncogenic role in breast cancer, such as FGFR1, has been described as amplified [38]. LSM1 is involved in pre-RNA splicing by acting on the removal of the 5′ cap structure [24,39,40]. *LSM1* has been studied in other tumor types, such as pancreatic cancer, observing a role in cancer progression, metastasis, and resistance to chemotherapies [41]. CLNS1A is involved in both the assembly of spliceosomal snRNPs and the methylation of Sm proteins [25,42]. CLNS1A cooperates with the protein PRMT5 and functions as an epigenetic activator of AR transcription in castration resistance prostate cancer [43]. *CLNS1A* has also been described in malignant gliomas [44], but data for breast cancer is very limited.

An interesting observation is the fact that the overexpression and amplification of these three genes was associated with detrimental prognosis in two large datasets, particularly in the luminal breast cancer subtype. Furthermore, the genomic knockdown of these transcripts reduced cell proliferation, suggesting an effect on cell growth.

Pharmacological modulation of transcription and RNA regulation is key for the optimal development of therapeutic strategies against key proteins. Spliceosome inhibitors have been developed, particularly those that bind to the HEAT repeats domain of some proteins, such as SF3B1 [45]. A comprehensive description has been recently reviewed elsewhere and beyond the scope of this article [12]. However, another approach to target this family of genes is the use of epigenetic modulators, such as BET inhibitors, to modulate transcriptional regulators or genes involved in RNA maturation. Examples have been provided with BET inhibitors, such as MK-8628 or ZEN003694 [12]. In this context, we observed that administration of the BET inhibitor JQ1 and the BET-PROTAC MZ1 reduced the expression of the three identified genes at different levels in two characteristic estrogen receptor breast cancer cell lines, MCF7 and T47D. In addition, these agents displayed a significant antiproliferative effect on these cell models. Although we agree that the antiproliferative effect of the compound could be multifactorial and the participation of these genes is a part and not a whole, we demonstrate in breast cancer that BETi can modulate the expression of splicing-related agents.

## 5. Conclusions

In conclusion, we describe novel splicing genes amplified in luminal breast tumors that are associated with detrimental prognosis and can be modulated pharmacologically. This data opens the door for further studies, confirming the effect of these genes in patients treated with BET inhibitors.

## Data Availability

All the data used in this study are available in public functional genomics data repositories (GEO, EGA, cBioportal, and TCGA).

## Supplementary Materials

The following are available online at https://www.mdpi.com/article/10.3390/cancers13164118/s1, Figure S1: Percentage of tumors with non-silent mutations for each splicing-related gene; Figure S2: Relation between expression level and CNAs for *ILF2*, *CLNS1A*, *LSM1,* and *ESRP1* genes; Figure S3: Associations of the transcriptomic fingerprint associated with amplification of *LSM1*, *CLNS1A,* and *ILF2* genes (genotype-2-outcome) and clinical outcome (OS and RFS); Figure S4: Presence of CNAs in our selected genes (*LSM1, CLNS1A,* and *ILF2*) in different tumor types (GDC Data Portal; 67 primary sites; Gain: red and Loss: blue); Table S1: List of splicing related genes evaluated in our study; Table S2: Percentage of tumors with mutation for each gene; Table S3: Common and specific splicing-related genes depending on molecular subtypes. Table shows the percentage of tumors with a high level of amplification for each gene (only included those with high amplification in >5%); Table S4: Prognostic value (RFS and OS) of the amplified genes (with a cutoff of >5% of tumors) in the HER2+ and Basal-like subtypes; Table S5: List of the transcripts included in the signatures associated with the CNA gain of *LSM1*, *CLNS1A,* and *ILF2*. Table S6: Univariate and Multivariate COX regression analysis to assess the potential prognostic value of CLNS1A, ILF2, and LSM1 expression in Luminal breast cancer patients.

## References

[1] Dvinge H., Kim E., Abdel-Wahab O., Bradley R.K. (2016). RNA splicing factors as oncoproteins and tumour suppressors. Nat. Rev. Cancer.

[2] Xu Y., Zhao W., Olson S.D., Prabhkara K.S., Zhou X. (2018). Alternative splicing links histone modifications to stem cell fate decision. Genome Biol..

[3] Djebali S., Davis C.A., Merkel A., Dobin A., Lassmann T., Mortazavi A., Tanzer A., Lagarde J., Lin W., Schlesinger F. (2012). Landscape of transcription in human cells. Nature.

[4] Kim E., Magen A., Ast G. (2007). Different levels of alternative splicing among eukaryotes. Nucleic Acids Res..

[5] Niklas K.J., Bondos S.E., Dunker A.K., Newman S.A. (2015). Rethinking gene regulatory networks in light of alternative splicing, intrinsically disordered protein domains, and post-translational modifications. Front. Cell Dev. Biol..

[6] Vohhodina J., Barros E.M., Savage A.L., Liberante F.G., Manti L., Bankhead P., Cosgrove N., Madden A.F., Harkin D.P., Savage K.I. (2017). The RNA processing factors THRAP3 and BCLAF1 promote the DNA damage response through selective mRNA splicing and nuclear export. Nucleic Acids Res..

[7] Mauger O., Scheiffele P. (2017). Beyond proteome diversity: Alternative splicing as a regulator of neuronal transcript dynamics. Curr. Opin. Neurobiol..

[8] Wang E., Aifantis I. (2020). RNA Splicing and Cancer. Trends Cancer.

[9] Wan R., Bai R., Shi Y. (2019). Molecular choreography of pre-mRNA splicing by the spliceosome. Curr. Opin. Struct. Biol..

[10] Urbanski L., Leclair N., Anczuków O. (2018). Alternative-splicing defects in cancer: Splicing regulators and their downstream targets, guiding the way to novel cancer therapeutics. WIREs RNA.

[11] El Marabti E., Younis I. (2018). The Cancer Spliceome: Reprograming of Alternative Splicing in Cancer. Front. Mol. Biosci..

[12] Bonnal S.C., López-Oreja I., Valcárcel J. (2020). Roles and mechanisms of alternative splicing in cancer—Implications for care. Nat. Rev. Clin. Oncol..

[13] Goodall G.J., Wickramasinghe V.O. (2021). RNA in cancer. Nat. Rev. Cancer.

[14] Fish L., Khoroshkin M., Navickas A., Garcia K., Culbertson B., Hänisch B., Zhang S., Nguyen H.C.B., Soto L.M., Dermit M. (2021). A prometastatic splicing program regulated by SNRPA1 interactions with structured RNA elements. Science.

[15] Seiler M., Peng S., Agrawal A.A., Palacino J., Teng T., Zhu P., Smith P.G., Buonamici S., Yu L., Cancer Genome Atlas Research Network (2018). Somatic Mutational Landscape of Splicing Factor Genes and Their Functional Consequences across 33 Cancer Types. Cell Rep..

[16] Xiping Z., Qingshan W., Shuai Z., Hongjian Y., Xiaowen D. (2017). A summary of relationships between alternative splicing and breast cancer. Oncotarget.

[17] Pereira B., Chin S.F., Rueda O.M., Vollan H.K., Provenzano E., Bardwell H.A., Pugh M., Jones L., Russell R., Sammut S.J. (2016). The somatic mutation profiles of 2433 breast cancers refines their genomic and transcriptomic landscapes. Nat. Commun..

[18] Brian W.D., Helmenstine E., Walsh N., Gondek L.P., Kelkar D.S., Read A., Natrajan R., Christenson E.S., Roman B., Das S. (2019). Hotspot SF3B1 mutations induce metabolic reprogramming and vulnerability to serine deprivation. J. Clin. Investig..

[19] Wang Y., Bernhardy A.J., Cruz C., Krais J.J., Nacson J., Nicolas E., Peri S., van der Gulden H., van der Heijden I., O’Brien S.W. (2016). The BRCA1-delta11q Alternative Splice Isoform Bypasses Germline Mutations and Promotes Therapeutic Resistance to PARP Inhibition and Cisplatin. Cancer Res..

[20] Koedoot E., Wolters L., van de Water B., Le Dévédec S.E. (2019). Splicing regulatory factors in breast cancer hallmarks and disease progression. Oncotarget.

[21] Tanaka I., Chakraborty A., Saulnier O., Benoit-Pilven C., Vacher S., Labiod D., Lam E.W.F., Bièche I., Delattre O., Pouzoulet F. (2020). ZRANB2 and SYF2-mediated splicing programs converging on ECT2 are involved in breast cancer cell resistance to doxorubicin. Nucleic Acids Res..

[22] Gabriel M., Delforge Y., Deward A., Habraken Y., Hennuy B., Piette J., Klinck R., Chabot B., Colige A., Lambert C. (2015). Role of the splicing factor SRSF4 in cisplatin-induced modifications of pre-mRNA splicing and apoptosis. BMC Cancer.

[23] Marchesini M., Ogoti Y., Fiorini E., Aktas Samur A., Nezi L., D’Anca M., Storti P., Samur M.K., Ganan-Gomez I., Fulciniti M.T. (2017). ILF2 Is a Regulator of RNA Splicing and DNA Damage Response in 1q21-Amplified Multiple Myeloma. Cancer Cell.

[24] Lobel J.H., Tibble R.W., Gross J.D. (2019). Pat1 activates late steps in mRNA decay by multiple mechanisms. Proc. Natl. Acad. Sci. USA.

[25] Guderian G., Peter C., Wiesner J., Sickmann A., Schulze-Osthoff K., Fischer U., Grimmler M. (2011). RioK1, a new interactor of protein arginine methyltransferase 5 (PRMT5), competes with pICln for binding and modulates PRMT5 complex composition and substrate specificity. J. Biol. Chem..

[26] Hegele A., Kamburov A., Grossmann A., Sourlis C., Wowro S., Weimann M., Will C.L., Pena V., Lührmann R., Stelzlet U. (2012). Dynamic protein-protein interaction wiring of the human spliceosome. Mol. Cell.

[27] Mermel C., Schumacher S., Hill B., Meyerson M.L., Beroukhim R., Getz G. (2011). GISTIC2.0 facilitates sensitive and confident localization of the targets of focal somatic copy-number alteration in human cancers. Genome Biol..

[28] Fuentes-Antrás J., Alcaraz-Sanabria A.L., Morafraile E.C., Noblejas-López M.D.M., Galán-Moya E.M., Baliu-Pique M., López-Cade I., García-Barberán V., Pérez-Segura P., Manzano A. (2021). Mapping of Genomic Vulnerabilities in the Post-Translational Ubiquitination, SUMOylation and Neddylation Machinery in Breast Cancer. Cancers.

[29] Nagy A., Munkacsy G., Győrffy B. (2021). Pancancer survival analysis of cancer hallmark genes. Sci. Rep..

[30] Pongor L., Kormos M., Hatzis C., Pusztai L., Szabó A., Győrffy B. (2015). A genome-wide approach to link genotype to OS by utilizing next generation sequencing and gene chip data of 6697 breast cancer patients. Genome Med..

[31] Ciriello G., Gatza M.L., Beck A.H., Wilkerson M.D., Rhie S.K., Pastore A., Zhang H., McLellan M., Yau C., Kandoth C. (2015). Comprehensive Molecular Portraits of Invasive Lobular Breast Cancer. Cell.

[32] Győrffy B., Lanczky A., Eklund A.C., Denkert C., Budczies J., Li Q., Szallasi Z. (2010). An online survival analysis tool to rapidly assess the effect of 22,277 genes on breast cancer prognosis using microarray data of 1809 patients. Breast Cancer Res. Treat..

[33] Ocaña A., Pandiella A. (2020). Proteolysis targeting chimeras (PROTACs) in cancer therapy. J. Exp. Clin. Cancer Res..

[34] Ken-Ichi F., Takaki I., Mizuki M., Masashi K., Seiji M. (2020). Regulating Divergent Transcriptomes through mRNA Splicing and Its Modulation Using Various Small Compounds. Int. J. Mol. Sci..

[35] Denichenko P., Mogilevsky M., Cléry A., Welte T., Biran J., Shimshon O., Barnabas G.D., Danan-Gotthold M., Kumar S., Yavin E. (2019). Specific inhibition of splicing factor activity by decoy RNA oligonucleotides. Nat. Commun..

[36] Wang E., Lu S.X., Pastore A., Chen X., Imig J., Chun-Wei Lee S., Hockemeyer K., Ghebrechristos Y.E., Yoshimi A., Inoue D. (2019). Targeting an RNA-Binding Protein Network in Acute Myeloid Leukemia. Cancer Cell.

[37] Pavlyukov M.S., Yu H., Bastola S., Minata M., Shender V.O., Lee Y., Zhang S., Wang J., Komarova S., Wang J. (2018). Apoptotic Cell-Derived Extracellular Vesicles Promote Malignancy of Glioblastoma Via Intercellular Transfer of Splicing Factors. Cancer Cell.

[38] Voutsadakis I.A. (2020). 8p11.23 Amplification in Breast Cancer: Molecular Characteristics, Prognosis and Targeted Therapy. J. Clin. Med..

[39] Kufel J., Bousquet-Antonelli C., Beggs J.D., Tollervey D. (2004). Nuclear Pre-mRNA Decapping and 5′ Degradation in Yeast Require the Lsm2-8p Complex. Mol. Cell Biol..

[40] Tharun S., He W., Mayes A.E., Lennertz P., Beggs J.D., Parker R. (2000). Yeast Sm-like proteins function in mRNA decapping and decay. Nature.

[41] Little E.C., Camp E.R., Wang C., Watson P.M., Watson D.K., Cole D.J. (2016). The CaSm (LSm1) oncogene promotes transformation, chemoresistance and metastasis of pancreatic cancer cells. Oncogenesis.

[42] Chari A., Golas M.M., Klingenhäger M., Neuenkirchen N., Sander B., Englbrecht C., Sickmann A., Stark H., Fischer U. (2008). An assembly chaperone collaborates with the SMN complex to generate spliceosomal SnRNPs. Cell.

[43] Beketova E., Fang S., Owens J.L., Liu S., Chen X., Zhang Q., Asberry A.M., Deng X., Malola J., Huang J. (2020). Protein Arginine Methyltransferase 5 Promotes pICln-Dependent Androgen Receptor Transcription in Castration-Resistant Prostate Cancer. Cancer Res..

[44] Braun C.J., Stanciu M., Boutz P.L., Patterson J.C., Calligaris D., Higuchi F., Neupane R., Fenoglio S., Cahill D.P., Wakimoto H. (2017). Coordinated Splicing of Regulatory Detained Introns within Oncogenic Transcripts Creates an Exploitable Vulnerability in Malignant Glioma. Cancer Cell.

[45] Read A., Natrajan R. (2018). Splicing dysregulation as a driver of breast cancer. Endocr. Relat. Cancer.

