# Supplementary material for: Genomic Mapping of Splicing-Related Genes Identify Amplifications in LSM1, CLNS1A, and ILF2 in Luminal Breast Cancer
Source: Cancers (Basel). 2021 Aug 16;13(16):4118. doi: 10.3390/cancers13164118 (PMC8391113; doi:10.3390/cancers13164118)
Supplement: Supplementary file 1 [file cancers-13-04118-s001.zip › Table S6.pdf]

Supplementary Table S6. Univariate and Multivariate COX regression analysis to assess the potential prognostic value of *CLNS1A*, *ILF2* and *LSM1* expression in Luminal breast cancer patients.

RFS	*CLNS1A*	*ILF2*	*LSM1*	CLNS1A+ILF2+LSM1
Univariate	8.5E-07	7.3E-07	7.8E-07	1.1E-06
Multivariate:				
vs nodal status	1.1E-02 vs 7.3E-07	3.2E-03 vs 7.5E-07	2.0E-05 vs 8.8E-07	1.05E-05 vs 6.7E-07
vs grade	0.12 vs 7.6E-07	0.64 vs 1E-06	1.8E-02 vs 9.5E-07	0.04 vs 1E-06
vs size	0.51 vs 4E-07	0.66 vs 5.9E-07	0.11 vs 4.9E-07	0.19 vs 4.7E-07
vs age	0.04 vs 0.57	0.05 vs 0.76	2.6E-03 vs 0.61	6.3E-03 vs 0.7

OS	*CLNS1A*	*ILF2*	*LSM1*	CLNS1A+ILF2+LSM1
Univariate	0.02	2.2E-03	6.3E-06	2.9E-06
Multivariate:				
vs nodal status	0.14 vs 5.6E-05	0.04 vs 3.2E-05	0.011 vs 5.2E-05	2.2E-04 vs 5.3E-05
vs grade	0.18 vs 3.5E-07	0.13 vs 4.4E-07	4.8E-04 vs 4.5E-07	2.9E-03 vs 4.4E-07
vs size	0.08 vs 8.9E-07	0.21 vs 3.6E-07	0.07 vs 3.7E-07	9.8E-03 vs 5.9E-07
vs age	0.06 vs 0.54	0.017 vs 0.44	7.6E-04 vs 0.54	8.6E-08 vs 0.39
