# Supplementary figures and images for: Genomic Mapping of Splicing-Related Genes Identify Amplifications in LSM1, CLNS1A, and ILF2 in Luminal Breast Cancer
Source: Cancers (Basel). 2021 Aug 16;13(16):4118. doi: 10.3390/cancers13164118 (PMC8391113; doi:10.3390/cancers13164118)

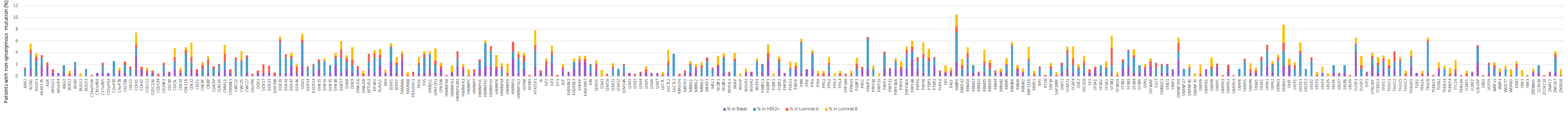

Supplement: Supplementary file 1 [file cancers-13-04118-s001.zip › Figure S1.png]

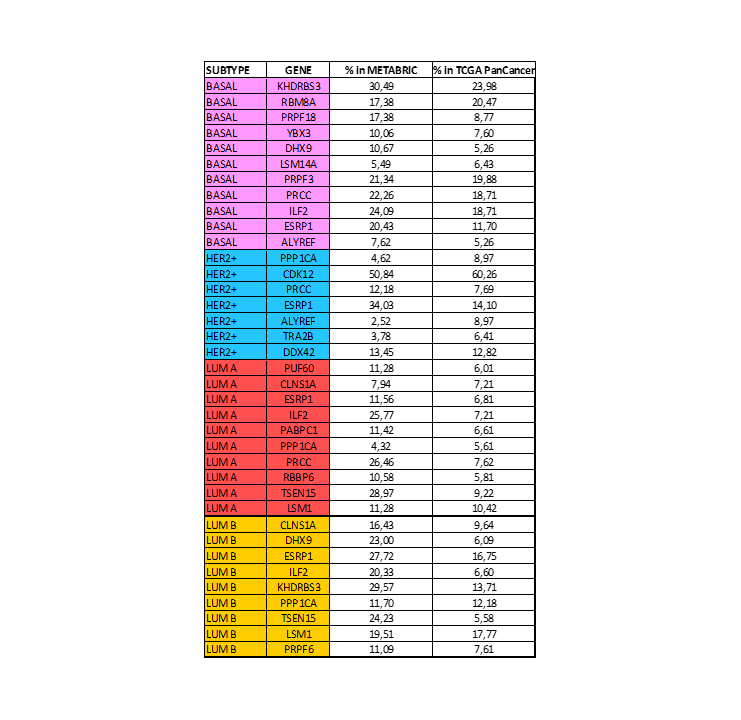

Supplement: Supplementary file 1 [file cancers-13-04118-s001.zip › Figure S2.TIF]

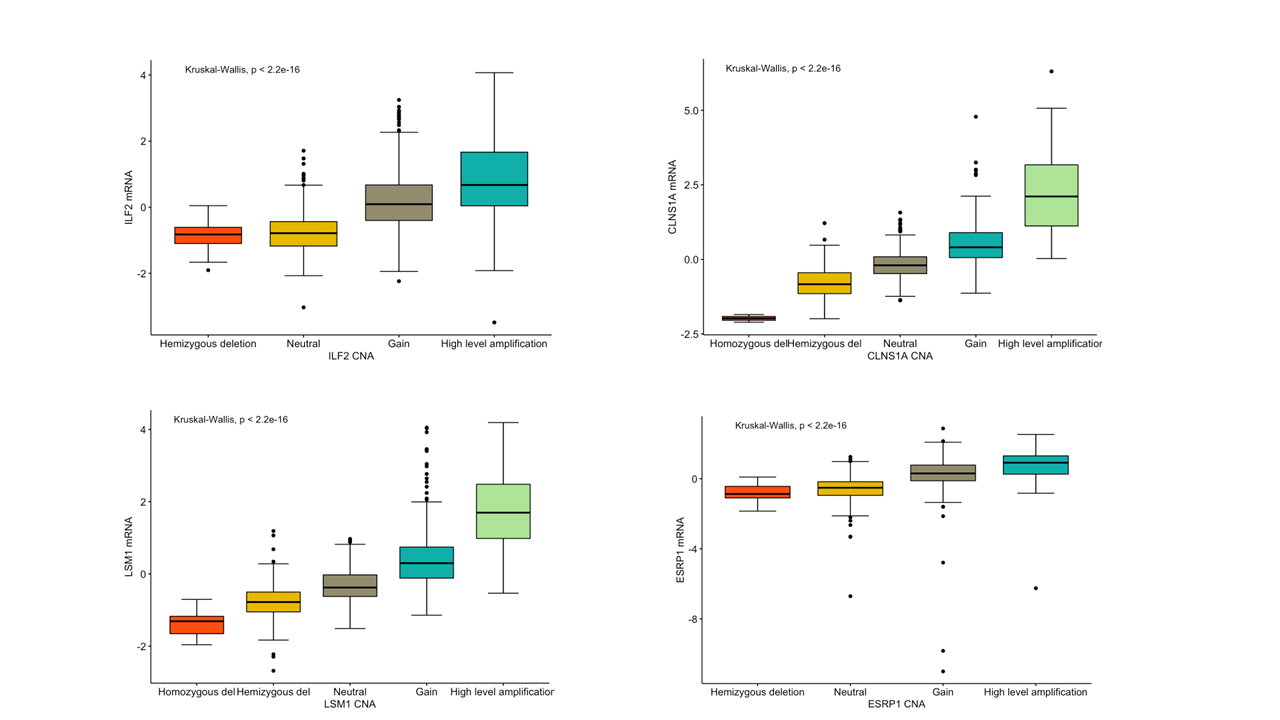

Supplement: Supplementary file 1 [file cancers-13-04118-s001.zip › Figure S3.tif]

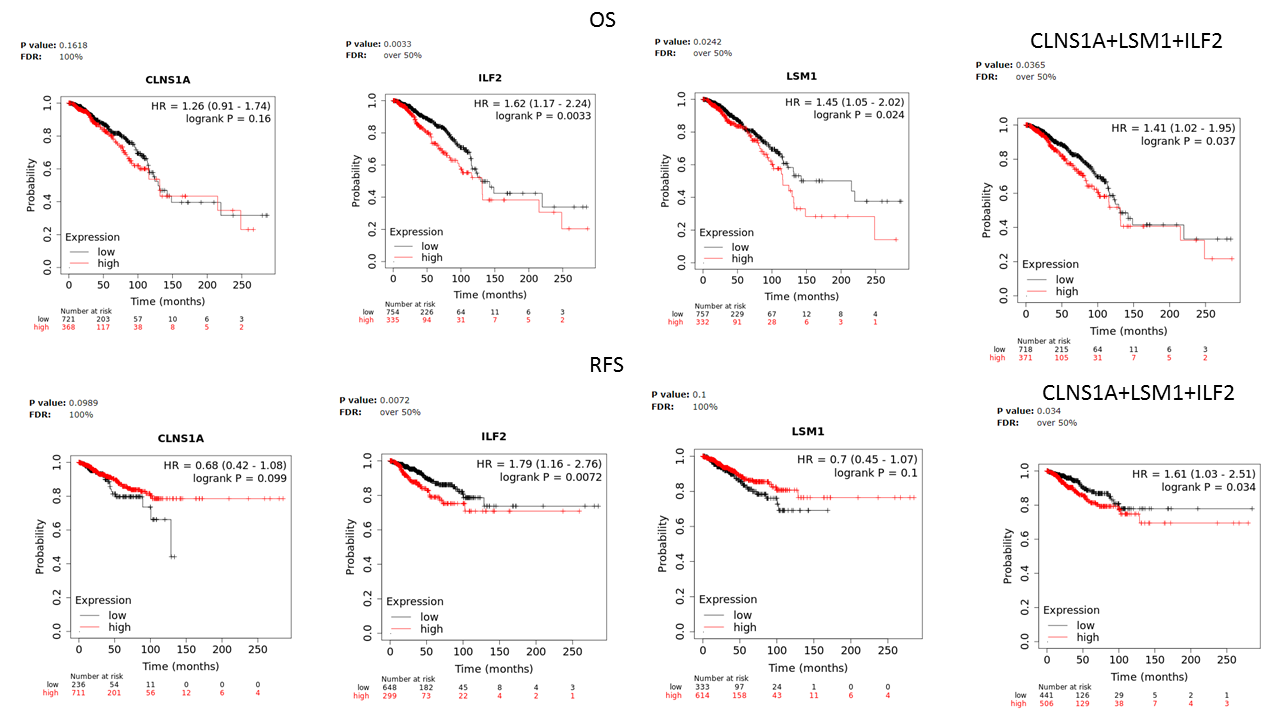

Supplement: Supplementary file 1 [file cancers-13-04118-s001.zip › Figure S4.tif]

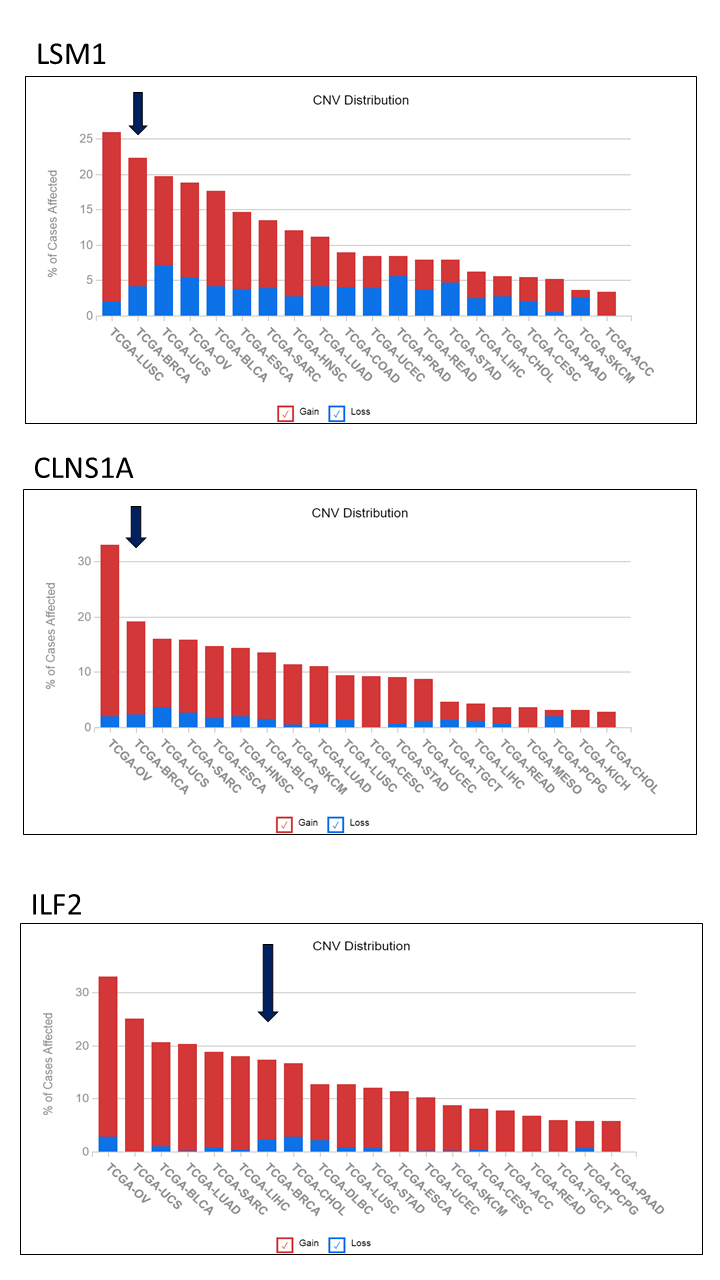

Supplement: Supplementary file 1 [file cancers-13-04118-s001.zip › Figure S5.tif]
